# Association of Common Polymorphisms in the Nicotinic Acetylcholine Receptor Alpha4 Subunit Gene with an Electrophysiological Endophenotype in a Large Population-Based Sample

**DOI:** 10.1371/journal.pone.0152984

**Published:** 2016-04-07

**Authors:** A. Mobascher, A. Diaz-Lacava, M. Wagner, J. Gallinat, T. F. Wienker, D. Drichel, T. Becker, M. Steffens, N. Dahmen, G. Gründer, N. Thürauf, F. Kiefer, J. Kornhuber, M. R. Toliat, H. Thiele, P. Nürnberg, O. Steinlein, G. Winterer

**Affiliations:** 1 Department of Psychiatry, Mainz University Hospital, Mainz, Germany; 2 Cologne Center for Genomics, University of Cologne, Cologne, Germany; 3 Department of Psychiatry, Bonn University Hospital, Bonn, Germany; 4 Department of Psychiatry and Psychotherapy, University Medical Center Hamburg Eppendorf (UKE), Hamburg, Germany; 5 Max-Planck Institute for Molecular Genetics, Berlin, Germany; 6 University of Greifswald, Greifswald, Germany; 7 Research Division, Federal Institute for Drugs and Medical Devices (BfArM), Bonn, Germany; 8 Department of Psychiatry, Rheinisch-Westfälische Technische Hochschule (RWTH) Aachen, University Hospital, Aachen, Germany; 9 Department of Psychiatry, Friedrich-Alexander University, University Hospital, Erlangen- Nürnberg, Erlangen, Germany; 10 Department of Addictive Behavior and Addiction Medicine, Central Institute of Mental Health, Mannheim, Germany; 11 Department of Human Genetics, Ludwig-Maximilians University, Munich, Germany; 12 Experimental and Clinical Research Center (ECRC), Charité – University Medicine, Berlin, Germany; Yale University, UNITED STATES

## Abstract

Variation in genes coding for nicotinic acetylcholine receptor (nAChR) subunits affect cognitive processes and may contribute to the genetic architecture of neuropsychiatric disorders. Single nucleotide polymorphisms (SNPs) in the *CHRNA4* gene that codes for the alpha4 subunit of alpha4/beta2-containing receptors have previously been implicated in aspects of (mostly visual) attention and smoking-related behavioral measures. Here we investigated the effects of six synonymous but functional *CHRNA4* exon 5 SNPs on the N100 event-related potential (ERP), an electrophysiological endophenotype elicited by a standard auditory oddball. A total of N = 1,705 subjects randomly selected from the general population were studied with electroencephalography (EEG) as part of the German Multicenter Study on nicotine addiction. Two of the six variants, rs1044396 and neighboring rs1044397, were significantly associated with N100 amplitude. This effect was pronounced in females where we also observed an effect on reaction time. Sequencing of the complete exon 5 region in the population sample excluded the existence of additional/functional variants that may be responsible for the observed effects. This is the first large-scale population-based study investigation the effects of *CHRNA4* SNPs on brain activity measures related to stimulus processing and attention. Our results provide further evidence that common synonymous *CHRNA4* exon 5 SNPs affect cognitive processes and suggest that they also play a role in the auditory system. As N100 amplitude reduction is considered a schizophrenia-related endophenotype the SNPs studied here may also be associated with schizophrenia outcome measures.

## Introduction

Various lines of basic, “translational” and clinical research suggest that the nicotinic cholinergic system modulates cognitive processes and that it plays an import role in a number of neuropsychiatric disorders such as schizophrenia, Alzheimer’s dementia, addiction and pain [1–4]. Therefore nicotinic acetylcholine receptors (nAchRs) are considered promising drug targets. NAchRs are ligand-gated ion channels consisting of 5 subunits surrounding a central pore that upon stimulation opens for influx of sodium and calcium ions into the neuron. The neurophysiological and pharmacological properties of NAchRs depend on the subunit composition that is highly variable [5]. The most abundant high-affinity NAchRs in the human brain, alpha4- and beta2-containing receptors, consist of two alpha4, two beta2 subunits and a fifth subunit that can be either alpha4, beta2 or a third kind of subunit [6]. Natural variation in *CHRNA4*, the gene coding for the alpha4 subunit, has been associated with a number of cognitive (endo)phenotypes and with aspects of nicotine dependence [7]. One “hot spot” for genetic variation is exon five of the *CHRNA4* gene where common synonymous single nucleotide polymorphisms (SNPs) have been the focus of previous research. In these previous studies addressing the role of SNPs in the exon 5 or other regions of the *CHRNA4* gene in human cognition or neuropsychiatric disorders have by and large either investigated the association of these SNPs with cognitive (endo)phenotypes such as electrophysiological or brain imaging measures [8, 9] in rather small numbers of subjects or with “clinical” measures such as smoking status and related outcome measures in larger cohorts [10, 11]. The existing literature on the role of rs1044396 in human cognition has recently been reviewed by Greenwood et al. [7]. The authors suggest that the *CHRNA4* rs1044396 variants affect attention and that the cognitive phenotype of this C/T SNP is characterized by greater ability of T allele carriers to preferentially process events in the attentional focus compared to events outside the attentional focus. However, in all of the previous electrophysiological or imaging studies including an own earlier functional magnetic resonance imaging (fMRI) study only a limited number of subjects were investigated and in most of theses studies the visual system was investigated using fMRI. Only one previous study investigated the impact of rs1044396 variants on auditory stimulus processing using electroencephalography (EEG) [12]. In this small-scale study subjects homozygous for the T allele exhibited higher auditory N1 amplitudes than carriers of the C allele. To our knowledge no replication of this study has been published thus far. Furthermore it has been argued that cognitive research suffers from an over-representation of certain population groups (i.e. college students) questioning the “generalizability” of the results [13]. In the present study we therefore sought to further investigate the impact of *CHRNA4* variants including rs1044396 on the ERP N1 component elicited by a “standard” two-stimulus auditory oddball task [14]. Here we applied the task to a large population-based sample of healthy German subjects [15]. Genotype effects on the N1 ERP were controlled for other variables such as smoking status and age.

## Subjects, Materials and Methods

### Study Sample

As part of the German Multicenter Study on Nicotine Dependence [15, 16] (www.nicotine-research.com), subjects were selected from the official local residents' registers and contacted by mail with the request to participate in the study. N = 49,000 subjects were contacted by letter, N = 4,140 subjects responded. With these subjects an initial 10-minute pre-screening was conducted on the phone. Those subjects who met the initial inclusion/exclusion criteria were invited for an in-depth screening investigation which included a standard medical examination, a psychiatric interview (SCID-1) [17], drug screening, a quantitative assessment of daily alcohol consumption and COHb measurements. Inclusion criteria: speaking German on mother-tongue level with grandparents of participants being born in Germany or an adjacent country. Furthermore, participants were required to be either current smokers (minimum one cigarette per day) or never-smoking individuals (no more than 20 cigarettes during their life). Currently abstinent smokers were not allowed to participate in the study. In order to confirm smoking status and to assess the degree of nicotine dependency, the Fagerstrom Test for Nicotine Dependence (FTND) was applied [18] and steady state exposure to cigarette smoke was assessed by measuring exhaled carbon monoxide (CO). Exclusion criteria: alcohol- or substance abuse within previous six months (DSM-IV), alcohol- or substance dependence (DSM-IV), other DSM-IV axis-1 psychiatric diagnosis during the last six months, serious impairments of vision/hearing, pregnancy, CNS-medication within previous six months, neurological illness (lifetime) or any other medical condition that may impact on brain function. From the 2,396 subjects that were investigated in the German Multicenter Study on Nicotine Dependence [15] (www.nicotine-research.com), *n* = 1,705 subjects were used for the present study since artifact-free EEG data and N100 peak amplitudes from electrode position Cz were available from these subjects (Table 1).

**Table 1:** Socio-demographic Data.

Parameter	Entire sample	TT	CT	CC
	N = 1705	N = 492	N = 846	N = 367
Age in years (SD)	35.3 (13.1)	34.7 (13.1)	35.2 (12.9)	36.4 (13.7)
% Females	56.8	53.7	58.6	56.9
Years of school education (SD)	11.7 (1.6)	11.8 (1.6)	11.7 (1.6)	11.7 (1.7)
% smokers	46.9	48.6	45.5	48
Exhaled CO in parts per million (SD)	6.8 (9.5)	7.5 (10.3)	6.3 (8.9)	6.9 (9.6)
FTND^#^ (SD)	3.1 (2.6)	3.0 (2.6)	3.0 (2.5)	3.4 (2.8)
Age of onset (years) ^#^ (SD)	16.1 (3.5)	16.3 (3.5)	16.0 (3.4)	16.0 (3.6)
Cigarettes per life time^§^ (SD)	6.4 (10.1)	6.9 (12.0)	6.0 (9.0)	6.9 (9.9)

^#^Only obtained in smokers.

^§^Only obtained in never-smokers.

The study was approved by the ethics committees of the local universities (Central Institute of Mental Health Mannheim, Charite Berlin, Friedrich-Alexander University Erlangen-Nürnberg, Heinrich-Heine University Duesseldorf; Rheinisch-Westfälische Technische Hochschule (RWTH) Aachen; University Hospital Bonn) at each study site and was conducted according to the declaration of Helsinki. Written informed consent was obtained from all participants. An identical standard operating procedure (SOP) and measurement time table was applied across all study sites including regular site monitoring and data quality controls. As part of the study, demographical and smoking-related information was obtained (Table 1). Electrophysiological experiments (see below) were conducted before a standardized lunch (between 11:30am and 02:00pm) and 1–3 hours after the last cigarette in smokers. This particular time window was selected to minimize acute nicotine as well as nicotine withdrawal-related effects. 45ml venous blood was taken for plasma cotinine measurements and genetic investigations (see below). For further details on study-related procedures see Lindenberg et al. [15].

### Electrophysiology/Functional Neuroimaging

*Task conditions*: Subjects had to keep their eyes closed during the EEG experiment. Firstly, five minutes of continuous “resting state” EEG was recorded. Immediately thereafter, EEG data was collected during an auditory oddball task—a reaction time task that requires a selective response to auditory stimuli. 240 auditory stimuli were presented binaurally by headphones at 70-dB sound pressure level and in (pseudo-)randomized order. Task instruction: to respond to target stimuli as quickly and accurately as possible (right-hand button press). Rare target stimulus: sinus tone of 2,000Hz (20% of stimuli), frequent non-target stimulus: sinus a tone of 1,500Hz (80% of stimuli) with stimulus duration of 50ms (including rise and fall time). Average inter-stimulus interval: 1,750ms (range 1,500–2,000ms).

*EEG data acquisition*: Across study sites, EEG recording was conducted with a 32-channel EEG system from Brain Products, Gilching, Germany (BrainAmp DC^®^, three study sites) or Neuroscan Inc. Sterling, VA, USA (Synamps 2^®^, four study sites). All study sites used the 2-channel EEG cap “EasyCap” (EEG Recording Caps and Products GmbH^®^, Breitbrunn, Germany). 29 scalp electrodes were placed according to the extended 10–20 system. Additional two electrodes were fixed at the outer canthi and above lower orbital portion of the orbicularis oculi muscle to detect horizontal and vertical eye movements. For recording the electrocardiogram (ECG), another electrode was attached to the left wrist. EEG was recorded relative to the Cz reference. Sampling rate of the analogue data: 250Hz. For N100 analysis (see below), a time constant of 0.1 sec. was preferred with a low-pass filter of 45Hz (both with slope of 12db). A 50Hz notch infinite impulse response (IIR) filter was used. Impedance was kept below 10kΩ for all electrodes.

*EEG data analysis*: Brain Vision Analyzer^®^ (Brain Products GmbH) was used for EEG analysis. Artifact detection was conducted semi-automatically and visually controlled. Voltage changes of more than 70μV were regarded as artifacts and excluded from further analysis. Average number of artifact-free and correctly responded target trials: 45.0 (SD 2.37). EEG data were then re-referenced to common average, segmented into 1,000ms epochs (-200ms to 800ms around target stimuli), corrected for baseline shifts and averaged. The event-related potential (ERP) N100 amplitude (μV) against baseline was calculated for the target condition (time window 90-110ms post stimulus). Remark: the ERP N100 was preferred instead of the ERP P300 because an earlier study conducted by Espeseth et al. [12] indicated a stronger rs1044396 genotype effect on N100.

For functional neuroimaging analyses, we performed current source density analyses of the event-related EEG (N100 amplitude, ±8 ms peak amplitude) in 3-D Talairach space using sLORETA [19, 20] following N100 analysis procedures previously described by us [21–23]. The “current source density” (μA/mm^3^) is defined as the divergence of the current density, i.e., the divergence is a spatial derivative. However, sLORETA is dimensionless, because it standardizes the current density (the denominator has the same units as the numerator), i.e., sLORETA units are simply units of standardized current density. The version of sLORETA employed uses the digitized Talairach atlas estimating the current density distribution of brain electrical activity on a dense grid of 6430 voxels at 5mm spatial resolution [20]. The solution space (the three dimensional space where the inverse problem is solved) is restricted to the cortical grey matter and hippocampus in the Talairach space. Only voxels significant at *P* = 0.01 or *P* = 0.05 level after correction for multiple comparisons are retained, i.e., the significance of changes in activity compared to baseline is assessed using non-parametric analyses adapted to source comparisons and implemented in sLORETA which corrects for multiple comparisons (statistical non-parametric mapping, SnPM) [24]. Standardized current density is depicted by co-registration of LORETA group maps with standard Talairach space and subsequently converted to a high-resolution MRI image.

### Genotyping

**Fig 1:**
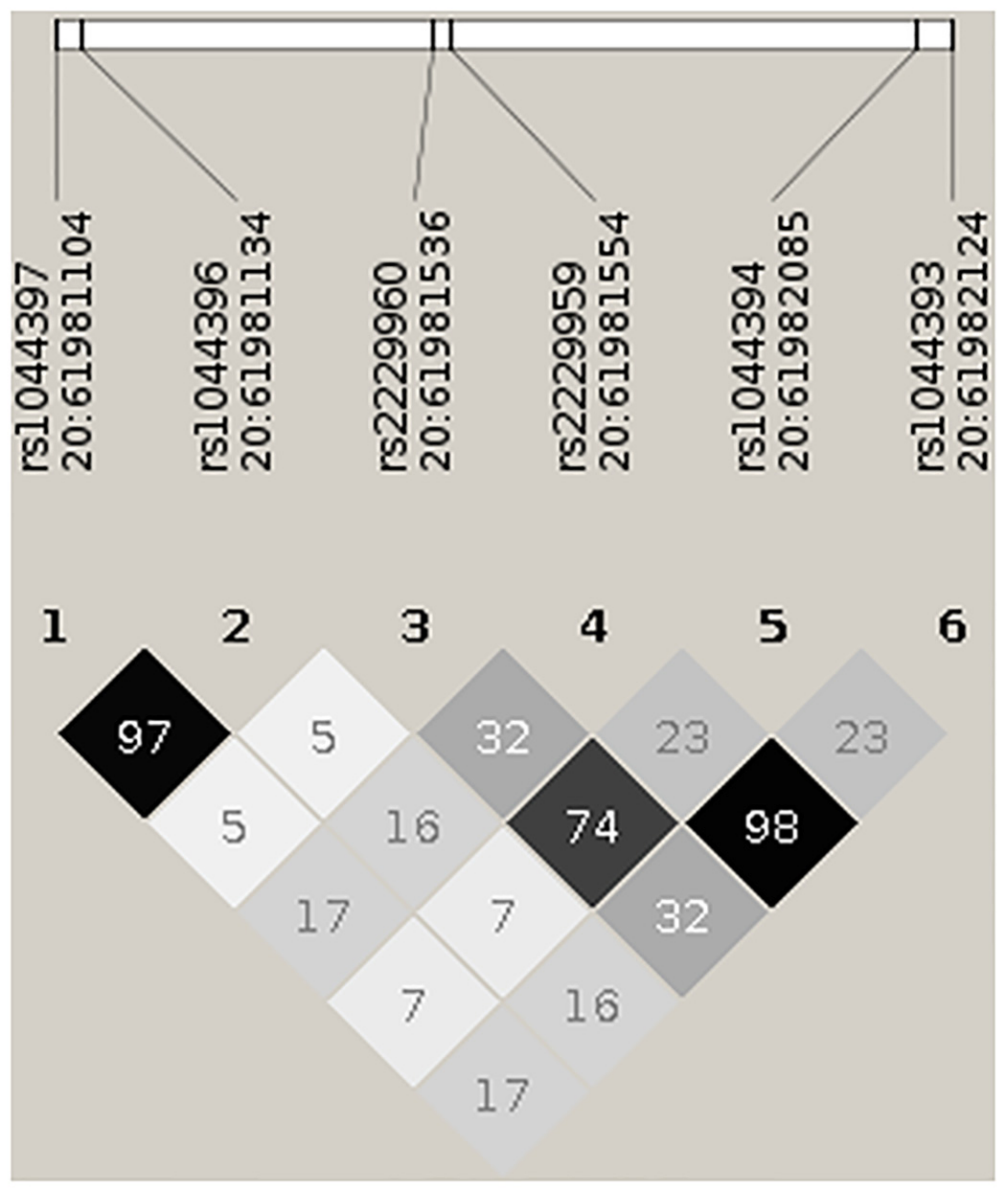
Pairwise correlation (r-squared) between the 6 SNPs on exon 5 of the CHRNA4 gene in our sample. The image was created using Haploview [43]. Genomic positions are given in hg19 coordinates.

Genotyping was performed at the Cologne Center for Genomics, University of Cologne blinded to phenotype status of the subjects. DNA was extracted from fresh frozen EDTA blood using the Qiagen FlexiGene DNA Kit according to the manufacturer's protocol. DNA quantification was done by using the NanoDrop Spectrophotometer (ND-1000, Thermo Fischer Scientific, Wilmington DE, USA) and we normalized the amount of DNA input based on RNase P copy number measurements using the TaqMan human RNase P assay (Applied Biosystems^®^, Foster City, CA, USA). Genotyping of the six *CHRNA4* (HGNC:1958) SNPs (5’-rs1044393, rs1044394, rs2229959, rs2229960, rs1044396, rs1044397-3’, see also Fig 1) which cover most of the *CHRNA4 exon 5* coding region, was performed with TaqMan SNP genotyping assays. These SNPs were selected based on previous research from our group [25] and others. Genotyping call rates were between 97.8% and 99.6%. Genotype frequencies at all six SNPs did not deviate from Hardy-Weinberg equilibrium (P > 0.05).

### Sequencing

A systematic analysis of the complete exon 5 of *CHRNA4* gene was performed in three overlapping fragments by polymerase chain reaction (PCR) and direct sequencing. The PCR primers were designed with the free internet tool Primer3 v.0.4.0 (http://bioinfo.ut.ee/primer3-0.4.0/). PCR products were purified by Exo/SAP digestion with Exonuclease I (New England Biolabs, Beverly, MA) and Shrimp Alkaline Phosphatase (Promega, San Diego, USA) and directly sequenced using the ABI PRISM BigDye^®^ Terminator v1.1 Cycle Sequencing Kit and the ABI 3730 sequencing instrument (Applied Biosystems, Foster City, CA, USA) following the manufacturer's instructions. All primer sequences and PCR conditions can be obtained on request from the authors. All sequences were analyzed using Mutation Surveyor software v3.2.

*Remark*: In an earlier paper, we have discussed the possibly of population stratification in our sample [16]. We did not genotype a standard panel of ancestry-informative SNPs to control for genetic stratification since we investigated a genetically highly homogeneous sample of subjects with Germany ancestry (or from adjacent countries) and because participants were randomly selected from the general population. Also, the European autosomal gene pool is quite small, in particular in northern and middle Europe populations [26].

### Statistical Analyses

For the primary analysis, a linear regression model with genotype predicting N100 amplitude was identified performing a stepwise backward selection with the statistical GLM Procedure (proc glm) implemented in SAS (SAS for Unix, version 9; SAS Institute Inc., Cary, NC, USA; http://www.sas.com). We performed best-fitting model selection based on an unconstrained genetic model. We included potentially modifying socio-demographic measures and smoking status as predictors (Table 2). Interactions among the predictors were tested considering up to 3-factor interactions. After the best model was selected, the recessive genetic model was evaluated based on previous literature [27] in addition to the unconstrained model. The best fitting model for the N100 ERP was used for testing the genotype effect on the corresponding behavioral measure, i.e., reaction time during oddball task condition (secondary analysis).

**Table 2:** Linear Regression Analyses: Variables.

Variable	Description	Data type^1^	Data range
Sex	subject gender	Cat	female, male
Age	subject age (years)	Quan	18–66
Center	sampling location	Cat	Aachen, Bonn, Berlin, Düsseldorf, Erlangen, Mainz, Mannheim
School	school attendance (years)	Quan	6–15
Smoker	smoking status	Cat	smoker, never-smoker
cig_day*	count of consumed cigarettes per day	Quan	0.25–70
Ftnd*	Fagerström Test "FTND" (score)	Ord	0–10
pack_years*	estimated sum of number of cigarettes packages consumed in life time (corrected for times of abstinence)	Quan	0.05–175
onset*	age of smoking onset (years)	Quan	6–44
Cohb*	COHb (exhaled CO in parts per million)	Quan	0–82
N100	N100 ERP amplitude μV (Cz) **	Quan	-1.58–11.95
rs1044396	Genotype	Cat	CC, CT, TT

List of variables evaluated by the stepwise backward selection in linear regression models.

^1^: cat = categorial, quan = quantitative, ord = ordinal.

*These variables were only included in the group of smokers. ERP = Event-related potential N100.

** Standardized scores.

Follow-up EEG-based sLORETA (N100) analyses: For between-group analyses, standardized current density are corrected for the effects of relevant covariates (age, gender, study site, education) before the residuals were entered into the final sLORETA analyses.

We finally addressed the question whether the 28 variants on exon 5 that were found in 1590 successfully sequenced samples were associated with N100 amplitude at electrode position Cz. Since all those variants had very low minor allele frequencies (MAF≤0.0054) we analyzed the combined set of variants using the established SKAT software package by Lee et al. [28] with default parameters and age, sex, smoking status and education as covariates. Both the burden test SKAT and the combined, “optimal” test SKAT-O resulted in non-significant p-values (p = 0.39 and p = 0.33, respectively). We conclude that in our sample, the N100 amplitude at electrode position Cz cannot be explained by association with rare variants.

## Results

### EEG Analyses

**Fig 2:**
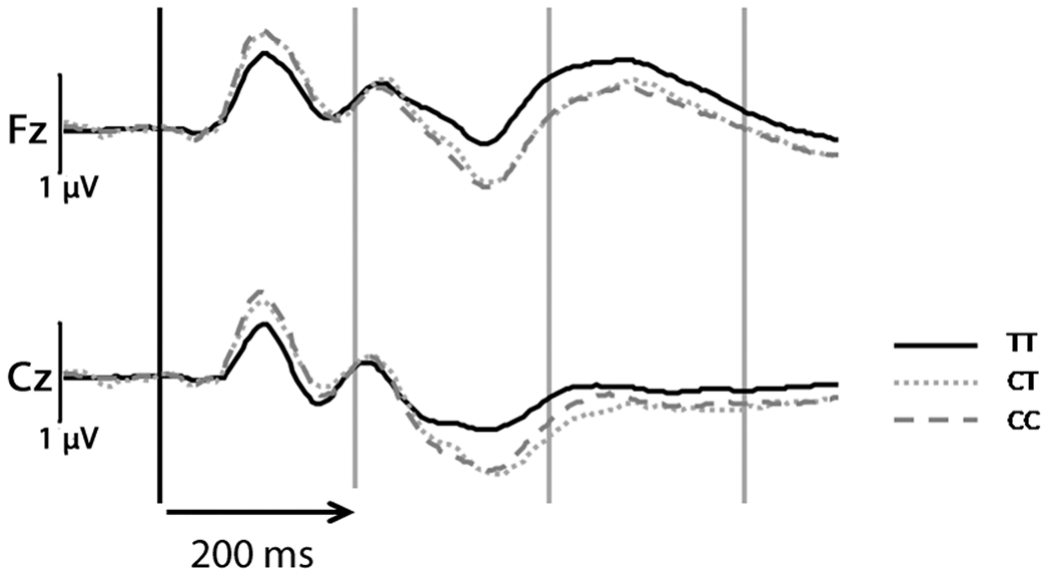
Event-related potentials (ERPs) incl. N100 during auditory oddball task (target responses) at electrode positions Fz and Cz for rs1044396 genotype groups. Note: response curves are uncorrected for covariates.

**Fig 3:**
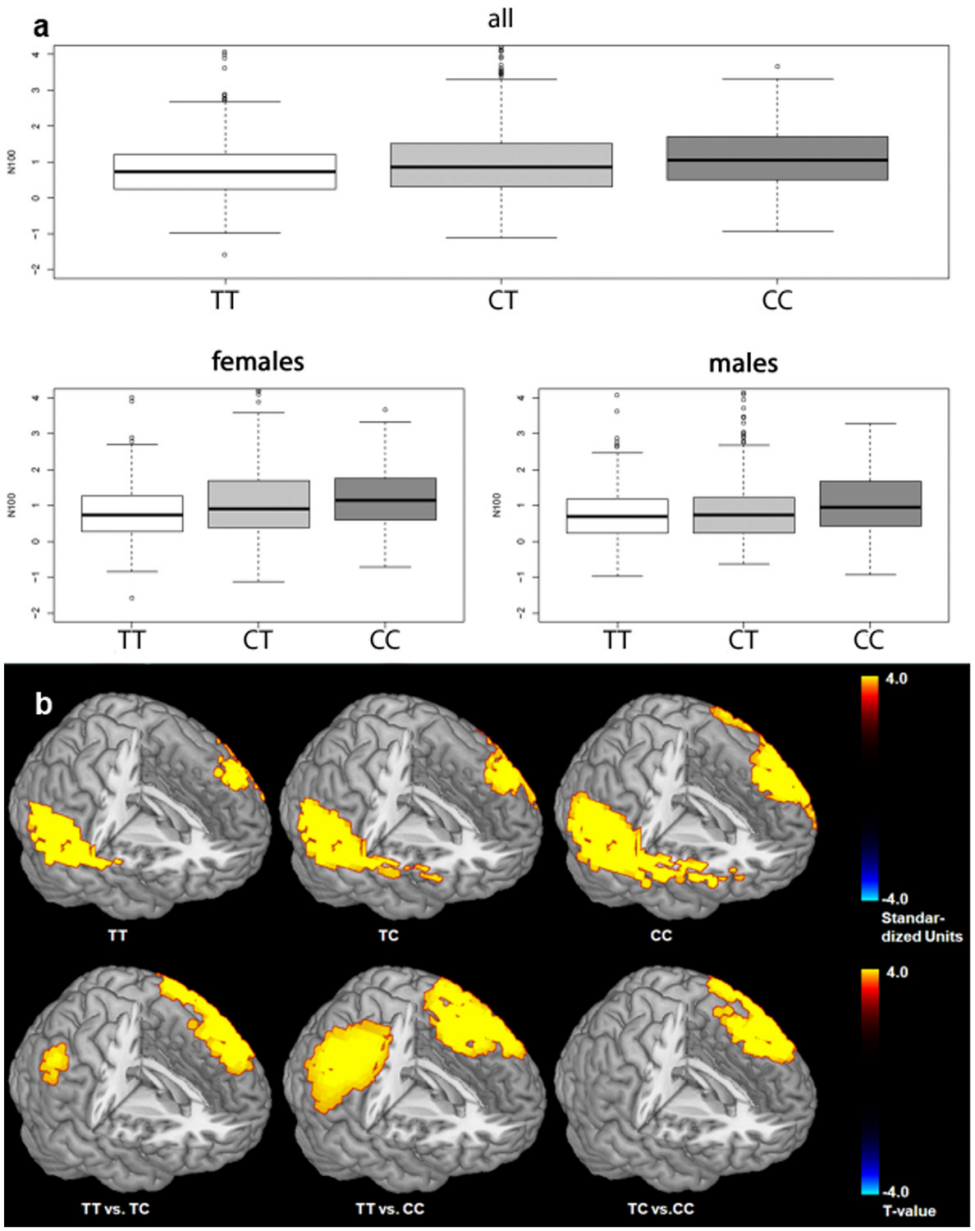
Quartile-quartile boxplot with genotype rs1044396 effects on event-related potential N100 amplitude (standardized values at electrode position Cz) in the general population. a) *Top*: Genotype effects in the entire sample (males and females). *Bottom*: Genotype effects separately depicted for males and females. Stepwise linear regression analysis of the event-related potential N100 at vertex electrode position Cz, with age, gender, education, smoking status and study site as covariates and testing for up to 3-factor interactions among the predictors, revealed a significant genotype effect (for details see results section). b) Functional neuroimaging sLORETA current density analyses of genotype groups with covariates age, gender, study site. *Top*: Current density for genotype groups (*P* < 0.01). *Bottom*: genotype group contrasts (t-values corrected for multiple testing). Independent of genotype, the strongest N100 activation maximum is seen in the left temporal lobe followed by a maximum in the frontal lobe mostly in the left hemisphere. This is consistent with intracortical recordings [44–46] as well as earlier LORETA studies conducted by us [21, 23] using comparable task conditions. For details on genotype effects see result section.

Among the six SNPs studied, stepwise linear regression including the covariates age, sex, smoking status and education revealed a significant effect of rs1044396 (genotype distribution: TT frequency 0.287, CT frequency: 0.497, CC frequency: 0.216) on N100-amplitude (F = 16.06, P < 0.0001). Mean amplitude values for the three rs1044396 genotype groups were: TT = 0.777 μV (SD 0.805), CT = 1.026 μV (SD 1.075) and CC = 1.093 μV (SD 0.821). See Table 3 for further details. A comparable association was obtained for rs1044397 but not for the remaining four SNPs. Homozygous rs1044396 C-allele carriers show the highest and homozygous T-allele carriers the lowest N100-amplitude (Figs 2 and 3a, Table 3), the effect is most pronounced in the left prefrontal and temporo-parietal cortex as revealed by current density source analyses of N100 (Tables 4 and 5, Fig 3b). Applying a recessive genotype model (TT vs. TC + CC), a significant genotype effect was observed (F = 30.46, P < 0.0001) (Table 3). Stronger genotype effects on N100 were seen in females (F = 13.60, P < 0.0001,; recessive model: F = 26.38, P < 0.0001) than in males (F = 3.77, P < 0.0235; recessive model: F = 6.87, P = 0.0089) (Fig 3a).

**Table 3:** Genotype Effects on N100 Event-Related Potential (Cz).

Parameter	DF	*F*-Value	*Original P-Value*	*Beta*
**Unconstrained Genetic Model**				
Sex	1	10.39	0.0013	*Females* 0.22
Study Center	6	26.89	<0.0001	-0.08 (AC)
				0.39 (BE)
				0.66 (BN)
				0.09 (DU)
				-0.24 (ER)
				-0.34 (MA)
				0.00 (MZ)
Genotype	2	16.06	<0.0001	-0.23 (TT)
				-0.07 (TC)
				0.00 (CC)
Sex*Genotype	2	1.58	0.2072	*Females*
				-0.18 (TT)
				-0.01 (TC)
				0.00 (CC)
***Recessive Genetic Model***				
Sex	1	6.12	0.0134	*Females* 0.21
Study Center	6	26.88	<0.0001	-0.08 (AC)
				0.38 (BE)
				0.66 (BN)
				0.09 (DU)
				-0.24 (ER)
				-0.35 (MA)
				0.00 (MZ)
Genotype	1	30.46	<0.0001	-0.18
Sex*Genotype	1	3.11	0.0782	*Females*-0.17

Linear Regression models after stepwise backward selection of variables (*n* = 1,705 subjects). *Top*: Unconstrained genetic model (DF = 11; *p* < .0001; R² = 0.11; *β* = 0.95). *Bottom*: Recessive genetic model (DF = 9; *p* < .0001; R² = 0.11; *β* = 0.90). AC = Aachen, BE = Berlin, BN = Bonn, DU = Düsseldorf, ER = Erlangen, MA = Mannheim, MZ = Mainz.

**Table 4:** sLORETA: N100 Brain Activation Maxima.

Region	x	y	Z	BA	T
**TT**					
Temporal Lobe	-15	-10	-12	28	2.97
Temporal Lobe	-15	-11	-16	34	2.95
Temporal Lobe	-20	-20	-12	35	2.87
Frontal Lobe	-5	4	-13	25	2.78
Temporal Lobe	-20	-29	-3	27	2.75
Cingulate Cortex	-15	-34	-7	30	2.73
Temporal Lobe	-20	-6	-29	36	2.70
Frontal Lobe	-5	13	-22	11	2.53
Temporal Lobe	-20	3	-34	38	2.50
Sub-lobar Insula	-40	-9	5	13	2.48
Parietal Lobe	-15	-44	-6	19	2.46
Temporal Lobe	-25	-2	-34	20	2.46
Temporal Lobe	-25	-40	-15	37	2.40
**TC**					
Temporal Lobe	-64	-9	5	22	3.01
Temporal Lobe	-64	-9	10	42	2.99
Frontal Lobe	-54	-9	10	43	2.93
Occipital Lobe	5	-93	-8	18	2.86
Temporal Lobe	-59	-5	-4	21	2.86
Anterior Cingulate	15	-7	46	24	2.82
Occipital Lobe	5	-92	-4	17	2.81
Temporal Lobe	-10	-34	2	27	2.75
Cingulate Cortex	-15	-34	-7	30	2.71
Frontal Lobe	59	6	27	9	2.70
Sub-lobar Insula	-45	-9	5	13	2.68
Temporal Lobe	-20	-25	-7	28	2.68
Temporal Lobe	-20	-20	-12	35	2.66
Frontal Lobe	-59	5	9	44	2.63
Temporal Lobe	-15	-11	-16	34	2.61
Parietal Lobe	50	-27	47	40	2.58
Parietal Lobe	-15	-44	-6	19	2.56
Temporal Lobe	-20	-39	-6	36	2.49
Temporal Lobe	-54	-19	6	41	2.49
Frontal Lobe	0	0	-4	25	2.45
Posterior Cingulate	5	-13	28	23	2.43
Frontal Lobe	10	-12	47	31	2.41
**CC**					
Temporal Lobe	-15	-10	-12	28	2.90
Temporal Lobe	-15	-11	-16	34	2.86
Temporal Lobe	-20	-20	-12	35	2.81
Temporal Lobe	-20	-29	-3	27	2.75
Frontal Lobe	0	0	-4	25	2.74
Cingulate Cortex	-15	-34	-7	30	2.72
Temporal Lobe	-20	-6	-29	36	2.58
Sub-lobar Insula	-40	-9	5	13	2.52
Parietal Lobe	-15	-44	-6	19	2.47
Temporal Lobe	-50	-9	5	22	2.43

N100 current density peak maxima as identified by sLORETA in genotype rs1044396 groups (TT, TC, TT) corrected for multiple testing with covariates age, gender and study site during auditory oddball task (target condition) with corresponding Montreal Neurological Institute (MNI) x y z coordinates of peak EEG-response (t-value) in sLORETA anatomical standard space. BA = Brodman Area.

**Table 5:** N100 sLORETA Genotype Group Comparisons.

Region	BA	X	Y	Z	T
**TT vs TC**					
Frontal Lobe	9	35	41	35	3.52
Frontal Lobe	8	35	36	40	3.38
Frontal Lobe	46	45	40	26	3.30
Frontal Lobe	10	35	45	25	3.21
Frontal Lobe	6	5	45	35	2.80
Anterior Cingulate	32	15	35	21	2.80
Frontal Lobe	45	54	29	3	2.74
Frontal Lobe	11	5	53	-11	2.65
Frontal Lobe	13	45	25	8	2.63
Frontal Lobe	47	54	29	-1	2.62
**TT vs CC**					
Frontal Lobe	9	-54	6	32	3.66
Frontal Lobe	6	-59	6	27	3.61
Frontal Lobe	44	-50	11	22	3.53
Frontal Lobe	45	-54	11	22	3.50
Frontal Lobe	10	-10	39	-6	3.38
Frontal Lobe	11	-5	48	-11	3.36
Frontal Lobe	4	-59	-4	23	3.36
Anterior Cingulate	24	-5	29	-1	3.35
Frontal Lobe	8	-50	7	41	3.28
Frontal Lobe	46	-45	21	22	3.24
Frontal Lobe	47	-20	29	-6	3.20
Frontal Lobe	32	-20	39	12	3.17
Frontal Lobe	25	5	28	-18	3.17
Frontal Lobe	43	-50	-4	14	3.11
Temporal Lobe	22	-64	-4	9	3.10
Frontal Lobe	13	-40	25	8	3.05
Anterior Cingulate	33	-5	20	17	3.04
Temporal Lobe	42	-59	-9	14	3.01
Parietal Lobe	2	-54	-18	29	2.88
Parietal Lobe	1	-64	-18	33	2.78
Parietal Lobe	40	-54	-23	29	2.68
Temporal Lobe	21	-59	0	-4	2.65
Temporal Lobe	41	-54	-19	10	2.63
Cingulate Gyrus	23	-5	-13	28	2.62
Temporal Lobe	38	-50	14	-9	2.60
**TC vs CC**					
Frontal Lobe	9	50	26	36	3.92
Frontal Lobe	8	50	17	41	3.74
Frontal Lobe	6	54	7	41	3.74
Frontal Lobe	45	59	11	22	3.44
Frontal Lobe	46	54	25	22	3.43
Frontal Lobe	44	59	15	18	3.24
Frontal Lobe	4	64	-8	28	2.96
Parietal Lobe	3	64	-9	23	2.82
Parietal Lobe	43	64	-9	19	2.62
Frontal Lobe	10	40	45	25	2.41

N100 current density peak maxima as identified by sLORETA in genotype rs1044396 group contrast analyses (TT vs TC, TT vs CC, TC vs CC) corrected for multiple testing with covariates age, gender and study site during auditory oddball task (target condition) with corresponding Montreal Neurological Institute (MNI) x y z coordinates of peak EEG-response (t-value) in sLORETA anatomical standard space. BA = Brodman Area.

Linear regression analyses using data on genetic variants obtained by complete exon 5 sequencing confirmed the association results for rs1044397 and rs1044396 (both: P < 10^−9^) with no additional common or rare single marker being significantly associated with N100 (genotype and sequencing data available upon request).

### Behavioral Results

Mean reaction time for the three rs1044396 genotype groups were: TT = 363.62 ms (SD 81.76), CT = 357.98 ms (SD 76.44) and CC = 364.37 ms (SD 79.41). In statistical analyses analogous to the EEG data set no main effect of genotype on reaction time was found (unconstrained model: F = 1.25, P = 0.2872; recessive model: F = 0.88, P = 0.41). However, a genotype*sex interaction effect was seen on reaction time (unspecified genetic model: F = 4.45, P = 0.0118; recessive genetic model: F = 8.05, P = 0.0046): In females the TT genotype was associated with higher reaction times while in males it was associated with lower reaction times. See Table 6 for further details. Significant genotype effects on reaction time were observed in females: TT = 370.41 ms (SD 84.98); CT = 354.38 ms (SD 75.52), CC = 364.75 ms (SD 82.25) (F = 5.31, P = 0.0051; recessive genetic model: F = 7.48, P = 0.0063). See also Table 7. Largely identical results were obtained for the neighboring SNP rs1044397.

**Table 6:** Genotype Effects on Reaction Time.

Parameter	DF	*F*-Value	*Original P-Value*	*Beta*
**Unconstrained Genetic Model**				
Sex	1	0.13	0.7165	*Females* -1.09
Study Center	6	10.73	< .0001	-17.14 (AC)
				-1.69 (BE)
				-4.33 (BN)
				-2.68 (DU)
				-4.05 (ER)
				33.43 (MA)
				0.00 (MZ)
Genotype	2	1.25	0.2872	-9.44 (TT)
				-1.88 (TC)
				0.00 (CC)
Sex*Genotype	2	4.45	0.0118	*Females*)
				16.55 (TT)
				-9.06 (TC)
				0.00 (CC
**Recessive Genetic Model**				
Sex	1	0.98	0.3222	*Females* -7.48
Study Center	6	10.66	< .0001	-17.28 (AC)
				-1.94 (BE)
				-4.59 (BN)
				-2.86 (DU)
				-3.91 (ER)
				33.20 (MA)
				0.00 (MZ)
Genotype	1	0.68	0.4100	-8.15
Sex*Genotype	1	8.05	0.0046	*Females* 22.95

Linear Regression models fitted for N100 (Cz) performing stepwise backward selection of variables. *Top*: Unconstrained genetic model (DF = 11; *p* < .0001; R² = 0.04; *β* = 362.5; *n* = 1,818). *Bottom*: Recessive genetic model (DF = 9; *p* < .0001; R² = 0.04; *β* = 361.3; *n* = 1,818). AC = Aachen, BE = Berlin, BN = Bonn, DU = Düsseldorf, ER = Erlangen, MA = Mannheim, MZ = Mainz. The larger sample size (compared to the sample size with the N100 regression analyses) is due to subjects with available reaction time data but without artifact-free EEG from the original sample.

**Table 7:** Genotype Effects on Reaction Time in Females.

Parameter	DF	*F*-Value	*Original P-Value*	*Beta*
**Unconstrained Genetic Model**				
Study Center	6	8.08	< .0001	-15.25 (AC)
				-1.55 (BE)
				-7.69 (BN)
				-2.69 (DU)
				-4.28 (ER)
				39.46 (MA)
				0.00 (MZ)
Genotype	2	5.31	0.0051	7.21 (TT)
				-11.03 (TC)
				0.00 (CC)
**Recessive Genetic Model**				
Study Center	6	8.01	< .0001	-15.56 (AC)
				-2.03 (BE)
				-8.16 (BN)
				-3.01 (DU)
				-4.07 (ER)
				39.09 (MA)
				0.00 (MZ)
Genotype	1	7.48	0.0063	14.97

Linear Regression models fitted for N100 (Cz) performing stepwise backward selection of variables. *Top*: Unconstrained genetic model (DF = 8; *p* < .0001; R² = 0.05; *β* = 360.5; *n* = 1,036). *Bottom*: Recessive genetic model (DF = 7; *p* < .0001; R² = 0.05; *β* = 352.9; *n* = 1,036). AC = Aachen, BE = Berlin, BN = Bonn, DU = Düsseldorf, ER = Erlangen, MA = Mannheim, MZ = Mainz. rs1044396 genotype = TT (frequency: 0.272), TC (frequency: 0.512), CC (frequency: 0.216).

## Discussion

We found an association of rs1044396 with N100 amplitude elicited by a standard auditory oddball paradigm with the C allele being associated with higher N100 amplitude, an effect that was pronounced in females. Furthermore there was a genotype*sex effect on reaction time in this task. Similar results were obtained for rs1044397 but not for the other 4 *CHRNA4* exon 5 SNPs studied.

Our results are consistent with previous literature in that we found an effect of common *CHRNA4* variants on cognition (see Greenwood et al. for a recent review) [7]. The results add to the existing literature in several ways. To our knowledge this is only the second study looking at *CHRNA4* exon 5 genotype effects on stimulus processing and attention outside the visual system [7]. Only Espeseth et al. [12] studied the effect of rs1044396 on processing of auditory stimuli with EEG event-related potentials in a small sample of 42 subjects distributed over the three genotypes (CC, CT, TT). Contrary to our results they found that TT homozygotes exhibited higher N100 amplitudes than carriers of the C allele. Sample bias, population heterogeneity and/or other differences in the composition of the two study samples may account for the different genotype effects found. In the Espeseth study the sample consisted of somewhat older subjects (mean age across genotype groups: 59.5–63.9 years) with above-average intelligence (mean IQ across genotype groups: 121 to 125). In our study (total sample) mean age across genotype groups ranged from 34.6 to 37.0 years, and mean IQ (assessed as verbal IQ by means of the MWT-B) [29] with 104 to 107 may also be considered more “normal”. These age and IQ related differences between the samples of the study published by Espeseth et al. [12] and our study may account for the differences in rs1044396 genotype effects on N100 amplitude. Second, the task design (two-stimulus standard oddball task in our study vs. oddball with additional distractor stimuli in the Espeseth study) was also non-identical. Though unlikely it cannot be ruled out, that task-related differences may also contribute to the divergent results. Third, though somewhat speculative, differences in the “genetic background” between the two study populations may also play a role. Gene-gene interactions between CHRNA4 and the dopamine system have been reported with regard to attention and response to nicotinic stimulation [30, 31]. As the dopaminergic input to (prefrontal) cortical areas is fine tuned by the cholinergic system (see Mobascher and Winterer, for a review) [1] the “dopamine system context” may affect the directionality of CHRNA4 genotype effects on cognition and neurophysiological measures.

The behavioral results (reaction time) however, are—at least in females—consistent with the effects of rs1044396 on processing speed in an earlier study reported by Reinvang et al. [27], in which TT homozygotes performed more slowly than C allele carriers. On the other hand, Schneider et al. [32] recently reported reduced processing speed associated with the rs1044396 T-allele across three different visual tasks (Stroop, negative priming and Posner task). Sex/gender effects were not reported in that study, though this mostly female (86%) sample was not suited to study sex by genotype interactions. However sex-specific effects of polymorphisms in the nicotinic system on cognition and behavior have been reported previously [33, 34]. A possible explanation for the diverging results between the study reported by Schneider et al., [32] and our study is the different sensory system that was stimulated (auditory vs. visual). Another explanation might be differences in study population (e.g. age). In any case the sample size and the population-based recruiting procedure are important strengths of the current study with regard the “generalizability” of our results [13]. However, this data set is consistent with the notion that common *CHRNA4* exon 5 SNPs have effects on stimulus processing and cognition beyond the visual system which has been more extensively studied in that regard [7]. Furthermore our electrophysiological “brain imaging” approach using LORETA to map genotype differences on the N100 ERP provides new insights into the effect of *CHRNA4* on regional “brain activation”. Greenwood et al. [7] suggest the temporo-parietal junction to be a critical region for *CHRNA4* genotype effects. Our data are by and large consistent with this notion but also point to more widespread effects including other cortical regions including prefrontal areas.

To our knowledge this is the first study investigating any naturally occurring genetic variation regarding its effect on the auditory N100 ERP in a population-based sample of this size. The auditory N1 is considered as an endophenotype for schizophrenia [35, 36], and has been shown to be substantially heritable (h = 0.4) [37]. Our finding of a *CHRNA4* rs1044396 T-allele associated N100 amplitude reduction in healthy subjects has two implications for future research. First, it provides further evidence that N100 amplitude reduction might be a useful phenotype in translational research and “outcome marker” for the development of drugs targeting the nicotinic system. Second, it suggests that genetic variation in exon 5 of *CHRNA4* might be associated with schizophrenia *per se* or perhaps schizophrenia-related outcome measures like treatment response. This hypothesis is by and large supported by the existing literature on rs1044396 effects on cognitive processes in which genotype differences in behavioral and brain activity measures were similar to the differences that can be found between patients and healthy controls [8, 9, 12, 38–42].

The results presented here do not provide further direct mechanistic inside in how the investigated *CHRNA4* SNPs (all noncoding) might exert their effect on cognition. This may be considered a limitation of this work. However, it is largely ruled-out by the sequencing data that unknown polymorphisms in the region are responsible for this effect and a recent paper from our consortium provides first insights in how these silent SNPs may affect neurobiology on the molecular and electrophysiological (cellular) level [25]. Our work lays the ground for future studies on how synonymous *CHRNA4* exon 5 SNPs affect cognitive processes and how they might be associated with neuropsychiatric disorders such as schizophrenia.

## Supporting Information

S1 File N100 Data. Provided are N100 amplitude values and information reg. study site, age, sex and rs1044396 genotype for each study participant.
(TXT)

S2 File Reaction Time Data. Provided are reaction times and information reg. study site, age, sex and rs1044396 genotype for each study participant.
(TXT)
